# eGFP tagged ZnO nanoparticles from glioblastoma targeting ferroptosis and MRP1 in head and neck squamous cell carcinoma

**DOI:** 10.1186/s11671-026-04895-x

**Published:** 2026-09-14

**Authors:** Salida Ali, Yu Li, Ontana Yotnarong, Chi Yao, Yongle Zhan, Ruochen Ma, Ruofan Shi, Zhiqiang Wang, Rong Na, Theeranan Tangthong

**Affiliations:** 1 Division of Urology, Department of Surgery, School of Clinical Medicine, LKS Faculty of Medicine, The University of Hong Kong, Hong Kong, China; 2 Department of Otolaryngology-Head and Neck Surgery, The Eighth Affiliated Hospital, Sun Yat-sen University, Shenzhen, 518033 China; 3 Nuclear Technology Research and Development Center, Thailand Institute of Nuclear Technology (Public Organization), Nakhon Nayok, Thailand; 4 Department of Urology, Ruijin Hospital, Shanghai Jiao Tong University School of Medicine, Shanghai, China

**Keywords:** ZnO spiky nanoparticles, Head and neck squamous cell carcinoma, Ferroptosis, Glutathione peroxidase 4, Reactive oxygen species

## Abstract

**Background:**

Head and neck squamous cell carcinoma (HNSCC) remain one of the most aggressive malignancies worldwide, necessitating the development of targeted therapeutic strategies. While zinc oxide (ZnO) nanostructures have shown promise in nanomedicine, the mechanistic influence of nanoparticle morphology on HNSCC remains poorly understood.

**Methods:**

In this study, we synthesized and characterized green-synthesized spherical ZnO nanoparticles (NPs) and spiky ZnO nanoparticles (SNPs) using total polyphenols (TP) as a reducing and stabilizing agent. To track cellular interactions, the nanostructures were further modified with glioblastoma-derived eGFP tags. The antitumor efficacy and underlying mechanisms were evaluated across multiple HNSCC cell lines (Fadu, TU212, and TU686) using 2D cultures, 3D multicellular spheroids, and cellular internalization assays.

**Results:**

Chemical characterization revealed distinct architectural shifts between the spherical and spiky morphologies. Both nanoparticle types demonstrated significant dose-dependent anticancer activity; however, ZnO SNPs exhibited superior internalization and more pronounced tumor suppression in 3D spheroid models. Notably, ZnO SNPs showed higher efficacy in the highly aggressive hypopharyngeal cancer line (Fadu) compared to laryngeal carcinoma lines (TU212 and TU686).

**Conclusion:**

Mechanistic investigations revealed that ZnO SNPs exert their antitumor effects by modulating reactive oxygen species (ROS) production, downregulating multidrug resistance-associated protein 1 (MRP1), and inhibiting glutathione peroxidase 4 (GPX4). This synergy triggers potent ROS-induced ferroptosis. Our findings suggest that the hierarchical morphology of ZnO SNPs enhances their therapeutic potential, offering a novel, targeted approach for the treatment of aggressive HNSCC.

**Graphical abstract:**

**Figure:**
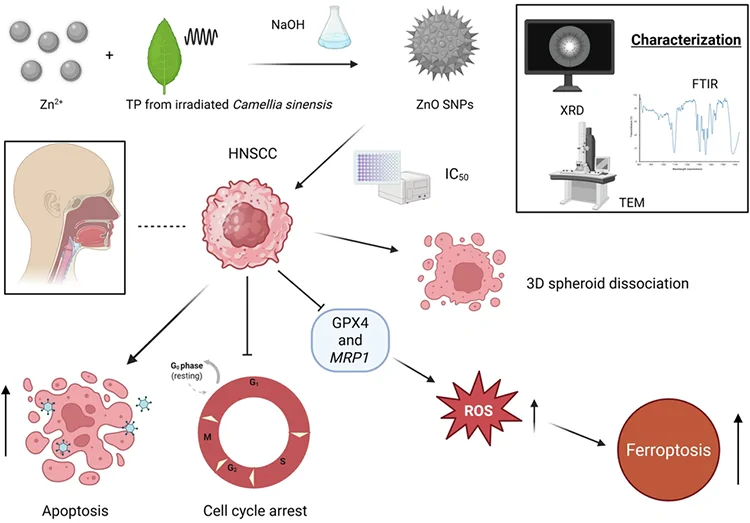

**Supplementary information:**

The online version contains supplementary material available at https://doi.org/10.1186/s11671-026-04895-x.

## Introduction

Head and neck squamous cell carcinoma (HNSCC) remains a global health challenge, characterized by high heterogeneity and significant morbidity and mortality rates [1, 2]. Despite advances in treatment, therapeutic resistance often limits clinical outcomes. Recently, ferroptosis—an iron-dependent form of regulated cell death driven by lipid peroxidation—has emerged as a promising strategy for limiting tumor progression [3, 4]. In cancer, reactive oxygen species (ROS) levels dictate ferroptosis sensitivity; while ROS can induce lethal lipid damage, cancer cells frequently upregulate antioxidant defenses to evade this process [5, 6]. Key regulators in this axis include glutathione peroxidase 4 (GPX4), which inhibits ferroptosis by mitigating ROS-induced damage [5], and multidrug resistance protein 1 (MRP1), encoded by ABCC1, which has been identified as a critical driver of multidrug resistance and a regulator of ferroptotic signaling [7]. Consequently, targeting the ROS/GPX4/MRP1 axis to induce ferroptosis represents a novel therapeutic frontier in HNSCC.

### Advancements in ZnO nanostructures and morphological significance

Nanomaterials have revolutionized oncology through their tunable physicochemical properties and targeted delivery capabilities [8, 9]. Among these, zinc oxide (ZnO) nanostructures have demonstrated potent, tumor-specific cytotoxicity by disrupting redox homeostasis and inducing ROS generation [10, 11]. While spherical ZnO nanoparticles (Zno NPs) have been widely characterized—specifically regarding caspase-3 activation and DNA fragmentation—there is a growing interest in anisotropic, non-spherical structures [12, 13]. Specifically, spiky ZnO nanoparticles (ZnO SNPs) offer unique advantages: their high-aspect-ratio topography enhances multivalent interactions with deformable cancer cell membranes, lowering the energetic barrier for endocytosis and promoting localized membrane disruption [14]. Furthermore, the increased surface area and reactive site density of ZnO SNPs accelerate intracellular Zn^2+^ release and ROS production, exploiting the impaired redox balance of malignant cells to achieve superior anti-tumor effects and deeper penetration into 3D tumor spheroids [13, 14].

### Synthesis and research gaps

To enhance biocompatibility and reduce off-target effects, synthesis methods utilizing plant extracts have gained traction. Polyphenols, such as those derived from green tea (TP) extract, serve as effective reducing agents that contribute intrinsic antioxidant and anti-cancer properties to the resulting nanoplatform [15–17]. However, most research on green-synthesized ZnO has focused on general cytotoxicity rather than detailed mechanistic pathways. To date, the mechanistic understanding of how anisotropic ZnO SNPs interact with ferroptosis-related modulators—specifically the GPX4 and MRP1 axis—remains largely unexplored in HNSCC.

### Study objectives

In this study, we developed and characterized ZnO NPs and ZnO SNPs using total polyphenol (TP) as a reducing agent. We aimed to evaluate the phenotypic changes and underlying molecular mechanisms of these nanoparticles in HNSCC cell lines. We hypothesize that the hierarchical spiky morphology of ZnO SNPs enhances ferroptosis-mediated tumor suppression by targeting the MRP1/ROS/GPX4 axis. Our findings offer a promising and practical approach for producing targeted ZnO SNPs as therapeutic agents, potentially improving efficacy and minimizing the adverse effects of traditional anticancer medications in HNSCC care.

## Experimental

### Synthesis and characterization of ZnO NPs and ZnO SNPs

Both ZnO NPs and ZnO SNPs were kindly given by our collaborator Dr. Theeranan Tangthong and her team from Nuclear Technology Research and Development Center, Thailand Institute of Nuclear Technology (Public Organization), Nakhon Nayok, Thailand. The detailed methods of synthesis and characterization were investigated. Briefly, we have conducted green synthesis of ZnO nanoparticles (ZnO NPs) using total phenols (TP) as reducing agent and stabilizing agent from green tea (*Camellia sinensis)*. Green synthesis of ZnO NPs were prepared by using the precursor of 0.2 M zinc acetate dihydrate ([Zn(CH₃COO)₂·2H₂O] solution mixed with the reducing agent of 1 mg/ml of TP solution at a ratio 1:1, and kept mixed-solution at room temperature for 9 h under dark conditions. Then, they chemically synthesized ZnO SNPs using zinc acetate dihydrate [Zn(CH₃COO)₂·2H₂O] and sodium hydroxide (NaOH) as precursor materials. 0.06 M of zinc acetate dihydrate [Zn(CH₃COO)₂·2H₂O] was dissolved in 50 mL distilled (DI) water, and 0.12 M NaOH was added slowly drop by drop with vigorous stirring. Then, the mixture solution was stirred for 5 h. Afterward, the aqueous precipitate was washed three times with deionized water, transferred to a crucible, and calcined at 450 °C for 5 h in an annealing furnace. The resulting ZnO SNPs powder was then collected and stored in a drying oven for subsequent use [14]. The characterization of ZnO NPs and ZnO SNPs were performed by Fourier Transform Infrared (FT-IR) spectroscopy, X-ray diffraction (XRD) measurements and Transmission electron microscopy (TEM) with a voltage of 120 kV. Both ZnO NPs and ZnO SNPs were diluted to different concentrations (0, 6.25, 12.5, 25, 50 and 100 μg/mL) before further cellular experiment.

### Extraction and purification of eGFP from glioblastoma cell line

The eGFP-tagged U87 was kindly given by Prof. Gilberto Leung from Department of Surgery, The University of Hong Kong. After the trypsinization of the cell, the cells were washed with PBS and the 10 ul of the proteinase and phosphatase inhibitor cocktail was added to the 990 ul of RIPA buffer to achieve 1X solution (on ice). Then, the cells were centrifuged at 15 000 × g for 15 min and the supernatant was collected and measured the protein concentration. The purification was done using Immobilized Metal Affinity Chromatography (IMAC) method which will selectively capture histidine-tagged proteins like EGFP. After that, the absorbance at 280 nm for protein and 488 nm for EGFP were used to quantify protein concentration and purity.

### Preparation of eGFP- ZnO NPs and ZnO SNPs

The 10 ul of aminopropyltriethoxysilane (APTES) in toluene was used to introduce amine group to ZnO NPs and ZnO SNPs. Then, the 10 ul of crosslinker EDC (1-ethyl-3-(3-dimethylaminopropyl) carbodiimide) and NHS (N-hydroxysuccinimide) were used to activate carboxyl groups of eGFP. This step was carried out in MES buffer at pH 5.5. Then, both eGFP and ZnO NPs and ZnO SNPs were mixed in room temperature overnight with constant stirring. Then, the confocal microscope was used to detect eGFP- ZnO NPs and ZnO SNPs.

### Cellular internalization of eGFP - ZnO NPs and ZnO SNPs in HNSCC cell lines

eGFP- ZnO NPs and ZnO SNPs were used to treat three HNSCC cell lines including laryngeal carcinoma cell lines (TU686 and TU212) and hypopharyngeal cancer cell line (Fadu) for 48 h and cellular internalization were observed using confocal Microscope LSM 880 with Airyscan 1. Clinical trial number: not applicable.

### 2D and 3D cell culture

Cellular phenotypes were conducted in three HNSCC cell lines including laryngeal carcinoma cell lines (TU686 and TU212) and hypopharyngeal cancer cell line (Fadu) (Chinese Academy of Sciences Cell Bank in Shanghai, China). Following the standard cell culture protocol and treatment with ZnO NPs and SNPs, Methyl thiazolyl tetrazolium (MTT) assay (Roche, Switzerland) was conducted in HNSCC cell lines to examine inhibitory effect and evaluate half-maximal inhibitory concentration (IC_50_). Then, following phenotypes were performed to evaluate the anti-tumor efficacy of ZnO NPs and SNPs (Supplementary Methods): (1) Cell cycle analysis (Cell Analyzer Agilent NovoCyte Quanteon and NovoExpress software); (2) Caspase-3 activity (Caspase-3 Activity Colorimetric Assay Kit, Elabscience, China) and (3) 3D spheroid size and morphology (Aggrewell^400^ 24 well plates, Stemcell Technologies).

### Ferroptosis pathway evaluation

After treated with ZnO NPs and ZnO SNPs, series of cellular phenotypes were performed to indirectly quantified ferroptosis pathway: (1) GPX4 activity was detected using a Glutathione Peroxidase 4 (GPX4) Activity Assay Kit (Elabscience, China) according to the manufacturer’s protocols. (2) The levels of intracellular ROS were examined using Reactive Oxygen Species Assay Kit (Beyotime, China). (3) Quantitative real‑time PCR (qRT‑PCR) of *MRP1* gene using a real-time quantitative PCR instrument according to product specification. GAPDH was used as an internal reference. (5) Lipid peroxidation was measured to further link the GPX4 activity and ferroptosis. The relative expression levels of genes were calculated as the method of the 2^−ΔΔCt^. The primers were shown in Table 1 in supplementary method.

### Inhibition of reactive oxygen species (ROS) using histamine dihydrochloride

Histamine dihydrochloride was used as an inhibitor of ROS production in HNSCC cell lines to evaluate if ZnO SNPs can regulate ROS production through GPX4. Then, GPX4 and ROS production were quantified according to the manufacturer’s protocols. To evaluate ability of ZnO SNPs to reverse ROS inhibition (Supplementary method).

### Western blot analysis

For the Western blot analysis of MPR1 and GPX4 expression in Fadu, TU686, and TU212 cell lines, cells were first lysed using RIPA buffer supplemented with protease and phosphatase inhibitors. Protein concentrations were determined using the BCA assay, and equal amounts of protein (typically 20–30 µg) were loaded onto 10%–12% SDS-PAGE gels for electrophoresis. Following separation, proteins were transferred onto PVDF membranes via wet transfer at 100 V for 1–2 h. The membranes were then blocked with 5% non-fat milk in TBS-T (Tris-buffered saline with 0.1% Tween-20) for 1 h at room temperature. Subsequently, the membranes were incubated overnight at 4°C with primary antibodies against MPR1, GPX4, and beta-actin (used as a loading control), each diluted according to the manufacturer’s instructions. After washing, membranes were incubated with appropriate HRP-conjugated secondary antibodies for 1 h at room temperature. Protein bands were visualized using an enhanced chemiluminescence (ECL) detection system, and images were captured for densitometric analysis. The expression levels of MPR1 and GPX4 were normalized to beta-actin to ensure equal loading and enable quantitative comparison across the cell lines.

### Lipid peroxidation assay

Lipid peroxidation levels were assessed using the malondialdehyde (MDA) assay to evaluate oxidative damage within the cells. Cells were first lysed and the supernatant was collected for analysis. The MDA levels were determined using a thiobarbituric acid reactive substances (TBARS) assay, in which MDA reacts with thiobarbituric acid (TBA) under acidic and high-temperature conditions to form a pink chromogen. The mixture was incubated at 95°C for 60 min, then cooled to room temperature, and the absorbance was measured at 532 nm using a spectrophotometer. A standard curve generated with known concentrations of MDA allowed for quantification of lipid peroxidation levels in the samples. Elevated MDA levels indicate increased lipid peroxidation and oxidative stress, providing insights into the extent of oxidative damage in the cell lines under study.

### In vivo validation

Male BALB/c nude mice (4–6 weeks) were maintained in an specific-pathogen-free (SPF) and access to food and water. The subcutaneous injection of FaDu with density of 2 × 106 in 100 ul suspension (80 ul basal RPMI1640 with 20 ul Matrigel) was performed. The oral administration of ZnO NPs and SNPs (2 times per week for 2 weeks) at dose of 2.5 mg/kg body weight was given after 7 days of injection. Tumor sizes were measured weekly. Then, mice were enthanized and tumor tissues and major organs (liver, kidneys, spleen, lungs) were collected for further mechanistic validation using Hematoxylin and Eosin Staining (H&E) for histopathological observation for toxicity. The intraperitoneal injection of overdosed phenobarbital was used as agent to euthanize animals. For the anesthesia, The intraperitoneal injection of 100 mg/kg with ketamine 10 mg/kg Xylazine. The anesthetic depth was verified via the pedal withdrawal reflex (toe pinch). The Balb/c nude mice were obtained from Zhuhai Bestest Biotechnology Co., Ltd. The mice were raised and maintained at Shenzhen Huarui Model Biotechnology Co., Ltd. The experimental protocol was approved by the Institutional Animal Care and Use Committee (IACUC) of Shenzhen Huarui Model Biotechnology Co., Ltd with HKU-SZ hospital (China); No. APS-260224-0038-001 in accordance with the Chinese National Standard Guideline for Ethical Review of Animal Welfare (GB/T 35892-2018).

### Statistical analyses

GraphPad software 10 (GraphPad Software, CA) was used as a statistical tool from at least three independent experiments (*n* = 3) with the mean ± standard deviation (SD). Unpaired two-tailed Student’s t test was used to analyze data between two groups, and differences among the multiple groups were assessed by one-way ANOVA and/or two-way ANOVA considering *p* value < 0.05 as statistically significant. (**** *p* < 0.0001; *** *p* < 0.001; ** *p* < 0.01; *p < 0.05; ns, no significant difference).

## Results and discussion

### Characterization of ZnO NPs and ZnO SNPs

The synthesis of metal oxide nanoparticles (NPs) has gained significant attention due to its environmental sustainability. In particular, total polyphenols (TP), which exhibit high antioxidant activity, can effectively act as reducing agents for Zn²⁺ ions during the green synthesis of ZnO nanoparticles [18, 19]. The total polyphenols present in the GT extract functioned as both reducing and stabilizing agents in the synthesis of ZnO NPs and ZnO SNPs, facilitating the formation of stable and crystalline nanoparticles without the use of chemical reducing agents. Moreover, the DPPH assay can be employed to infer the reducing capacity of polyphenols, especially in the context of nanoparticle synthesis. In this study, the antioxidant activity of TP was evaluated, and the results showed that the TP extract exhibited a DPPH radical scavenging activity of 69.7%.

The synthesis of spherical ZnO NPs using TP follows a biomolecule-mediated growth mechanism. The TP act as both a reducing and capping agent, forming a stable complex with Zn^2+^ ions. The formation of spiky ZnO nanoparticles using NaOH follows a three-step process: Nucleation: NaOH reacts with Zinc ions to form Zn(CH₃COO)₂·2H₂O complexes, which dehydrate into ZnO seeds. Core Formation: These seeds aggregate to form a central stable core.Anisotropic Growth: Due to the high surface energy of the (0001) crystal plane, the growth units from the NaOH solution preferentially deposit along the c-axis. This leads to rapid outward growth, resulting in the spiky morphology.

The synthesis of ZnO NPs and SNPs was characterized via UV-Vis spectroscopy (Supplementary Figure 1). The growth of the ZnO NPs was monitored by investigating the Surface Plasmon Resonance (SPR), which generates a prominent absorption peak. Consequently, UV-Vis spectra were recorded in the 200–600 nm range to track nanoparticle formation within the colloidal solution. The synthesis of ZnO NPs using TP extract is evidenced by a shift in absorption bands; the initial maximum absorption of the TP extract at 273 nm disappeared upon the formation of the nanoparticles, giving rise to a new characteristic peak at 316 nm (Supplementary Figure 1(A)). Furthermore, the spectra for the ZnO SNPs exhibited a distinct peak at approximately 530 nm, confirming the successful synthesis of both the ZnO SNPs components (Supplementary Figure 1(B)). The hydrodynamic particle size of the ZnO NPs and SNPs synthesized were 231.7 ± 20.42 nm and 2.15 ± 0.56 μm, respectively. Moreover, the surface charge of the ZnO NPs and SNPs synthesized were −3.29 ± 0.26 mV and −5.26 ± 0.11 mV, respectively.

**Fig. 1:**
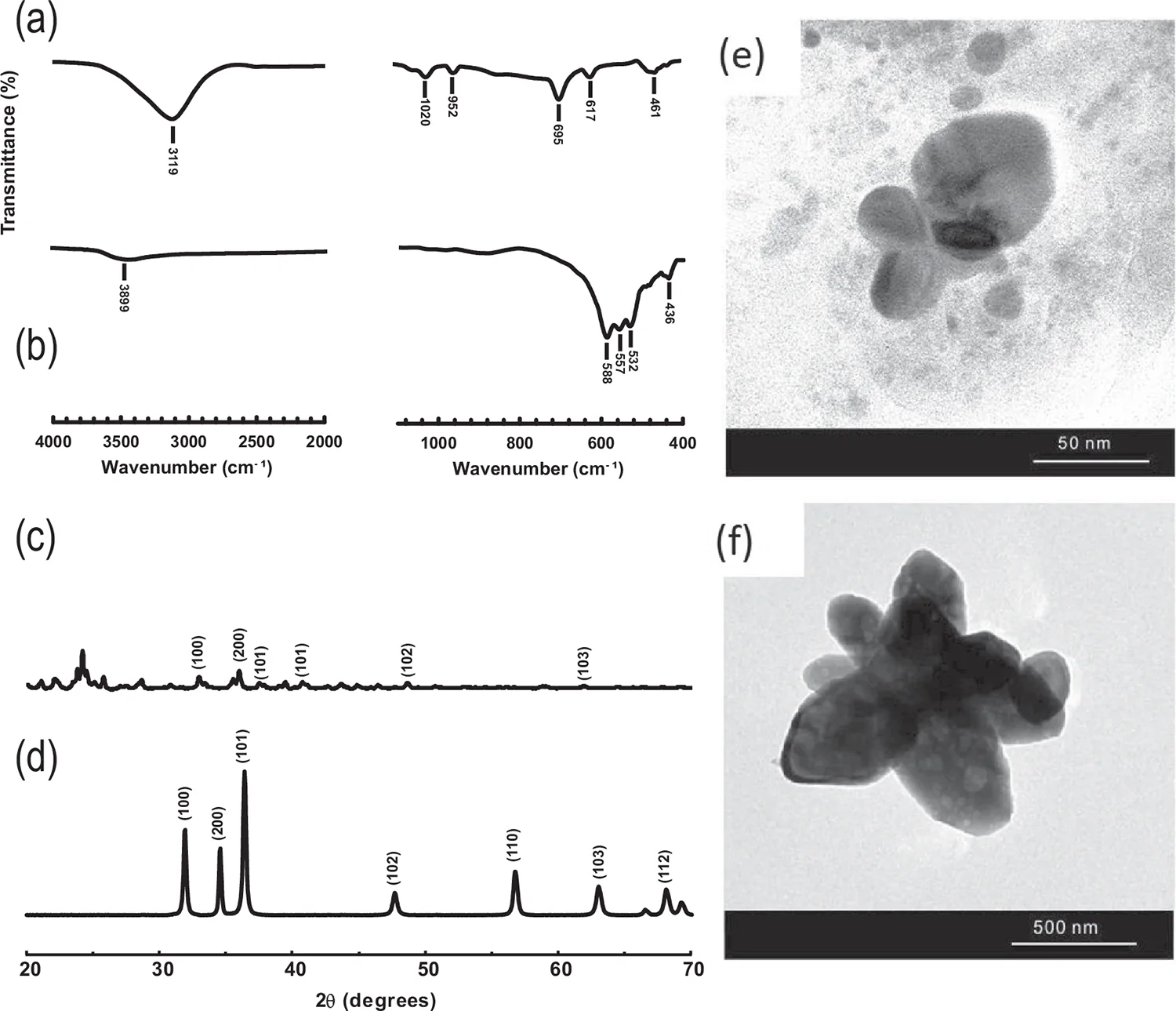
Characterization of ZnO NPs and ZnO SNPs. **a,b** FTIR spectra of ZnO NPs characterization illustrating distinct functional groups in ZnO NPs and ZnO SNPs respectively. **c,d** X-ray diffraction (XRD) showing crystalline patterns of ZnO NPs and ZnO SNPs. **e, f** Representative of Transmission Electron Microscopy (TEM) images depicting spherical and spiky morphology of ZnO NPs and ZnO SNPs characterization. Scale bar = 50 nm and 100 nm respectively

ZnO SNPs exhibited shifts in nano-architectures compared to ZnO NPs in characterization. FTIR revealed that there are shifts in major peaks and absorption bands in ZnO NPs (Fig. 1a) indicate compared to those of ZnO SNPs (Fig. 1b) pointing a change in the crystal structure or particle size from various synthesis conditions such as C-H stretching (3119) and O-H (3899). Figures 1c and 1d illustrated the X-ray diffraction (XRD) patterns for ZnO NPs and ZnO SNPs showing characteristic diffraction peaks in the crystal planes. Significantly sharper and more intense were observed in ZnO SNPs pattern at 100 and 101 planes hinting higher crystallinity, crystallite sizes and less amorphous content. TEM images shown in Figs. 1e and 1f illustrated the different in structural architecture of ZnO NPs (50 nm) and ZnO SNPs (200 nm) where ZnO NPs were more spherical, uniformed and isotropic compared to spike-like architecture and anisotropic growth in ZnO SNPs.

**Fig. 2:**
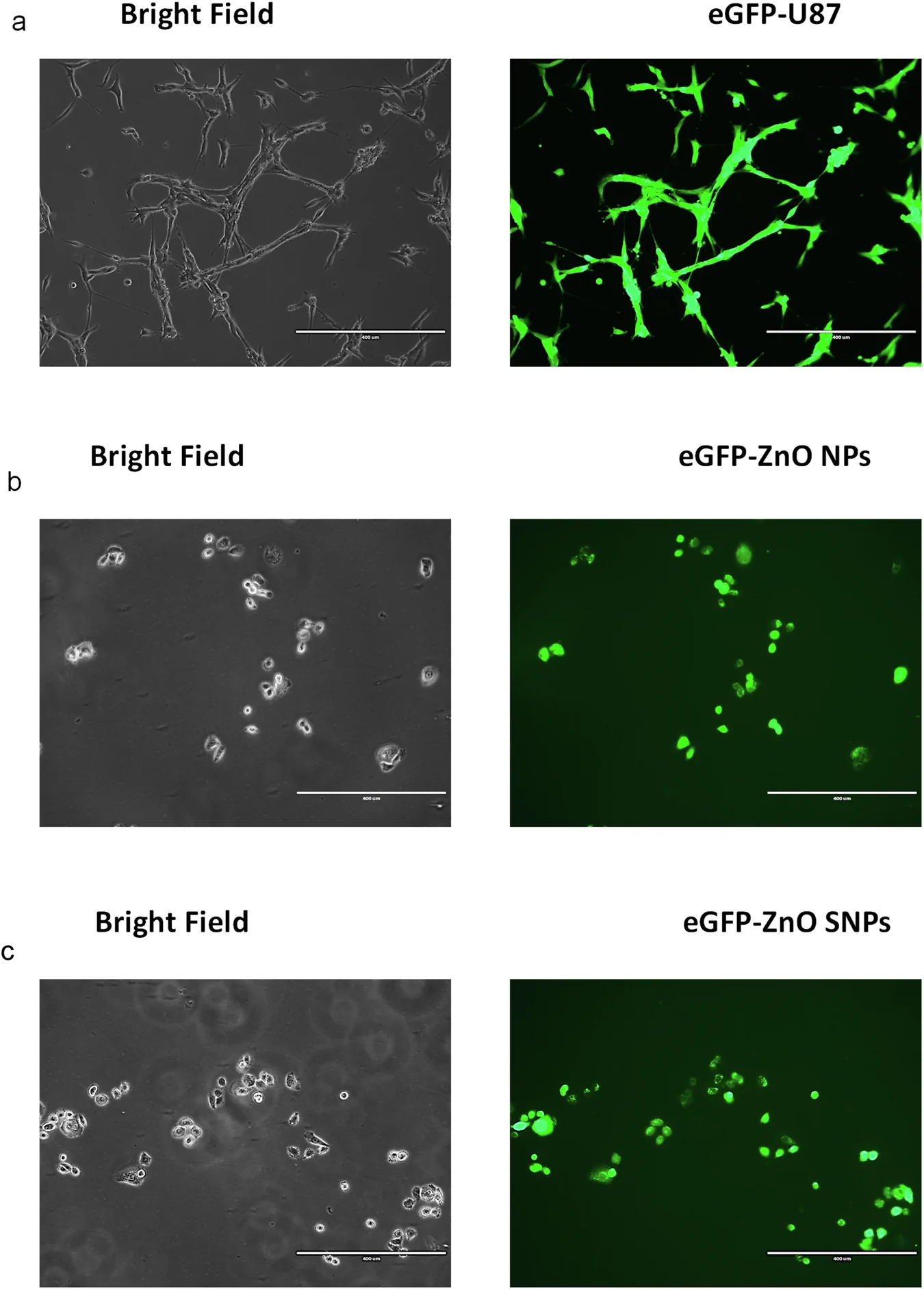
The characterization of eGFP-ZnO NPs and SNPs purified from U87-eGFP cell line. Presence of U87-eGFP morphology **a**, eGFP-ZnO NPs (**b)** and SNPs **c**. Cells were imaged using confocal microscopy

**Fig. 3:**
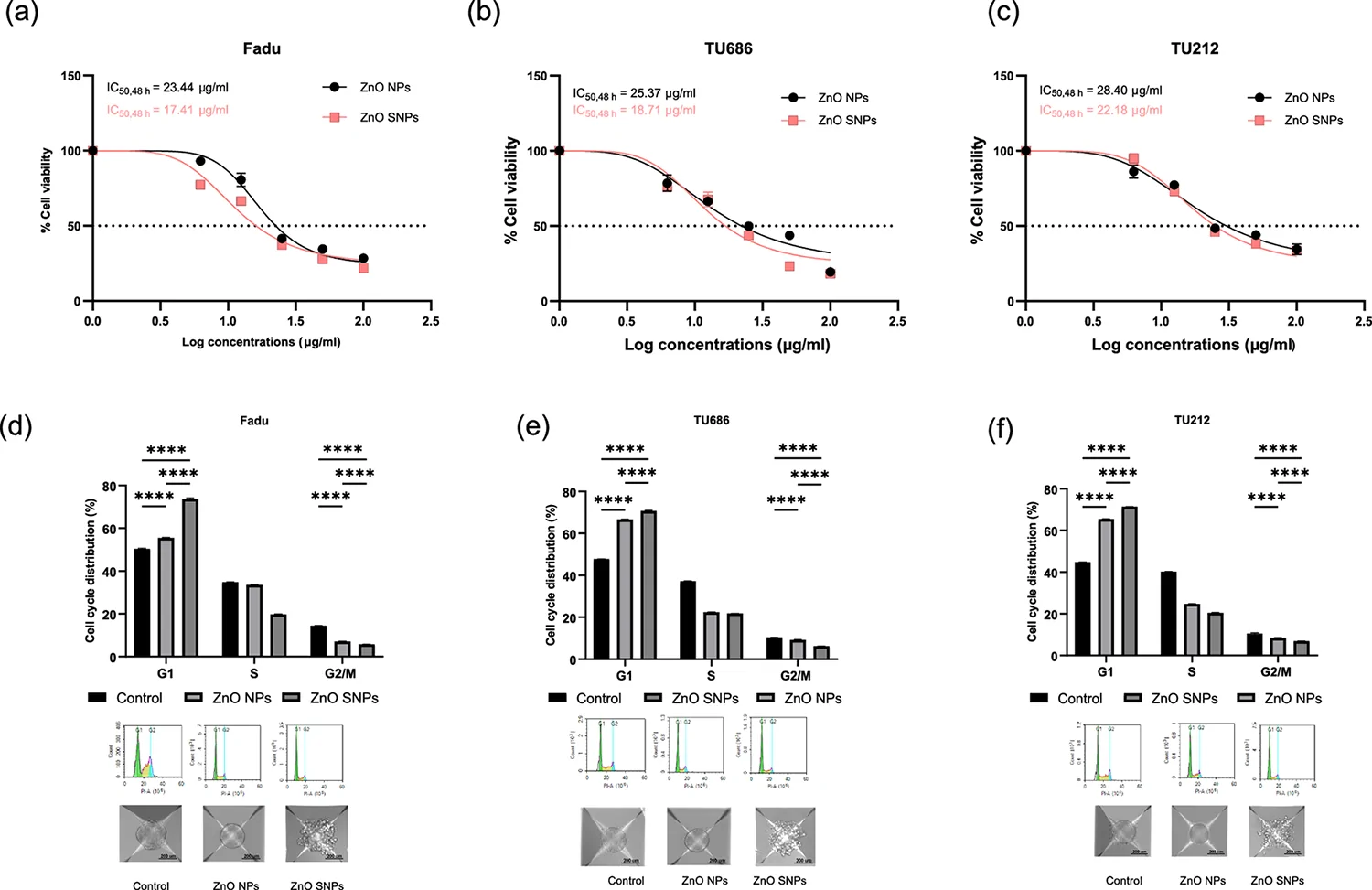
In vitro validation of anti-efficacy of ZnO NPs and ZnO SNPs in HNSCC cell lines. Fadu **a**, TU686 (**b**) and TU212 (**c**) were treated with different concentrations of ZnO NPs and ZnO SNPs for 48 hr. Data are represented as mean ± SD. Cell cycle analysis of HNSCC cell lines. The representative images and analyses of cell cycle analysis by flow cytometry of Fadu **d**, TU686 (**e**) and TU212 (**f**) after treated with ZnO NPs and ZnO SNPs or 48 h. All data are expressed as the mean ± standard deviation (SD) from three independent experiments (*n* = 3). The 3D structural morphology of Fadu **d**, TU686 (**e**) and TU212 (**f**) using Aggrewell^400^ after 48 h of ZnO NPs and ZnO SNPs treatment. The scale bar of the images is 200 µm. Two-way ANOVA was used to determine the significance of the comparisons of data (**** *p* < 0.0001; *** *p* < 0.001; ** *p* < 0.01; *p < 0.05; ns, no significant difference)

The successful synthesis and characterization of glioblastoma-derived eGFP tagged ZnO nanoparticles and ZnO spiky nanoparticles were revealed in Fig. 2. Both ZnO NPs and ZnO SNPs revealed the anticancer efficacy against 3 HNSCC cell lines compared to the control group with more effective dose-dependent manner in aggressive HNSCC cell line (Fadu) after 48 h of treatment (Fig. 3). For ZnO NPs, IC_50_ were 23.44, 25.37 and 28.40 µg/mL for TU212, TU686 and Fadu showing level of response from less aggressive to most aggressive cell line accordingly (Fig. 3a–c). IC_50_ of ZnO SNPs values were significantly lower in all cell lines showing the effect across HNSCC cell lines with the highest inhibitory effect on hypopharyngeal cancer cell lines (17.41, 18.71 and 22.18 µg/mL for TU212, TU686 and Fadu respectively, all *p* values < 0.05) compared to control and ZnO NPs.

**Fig. 4:**
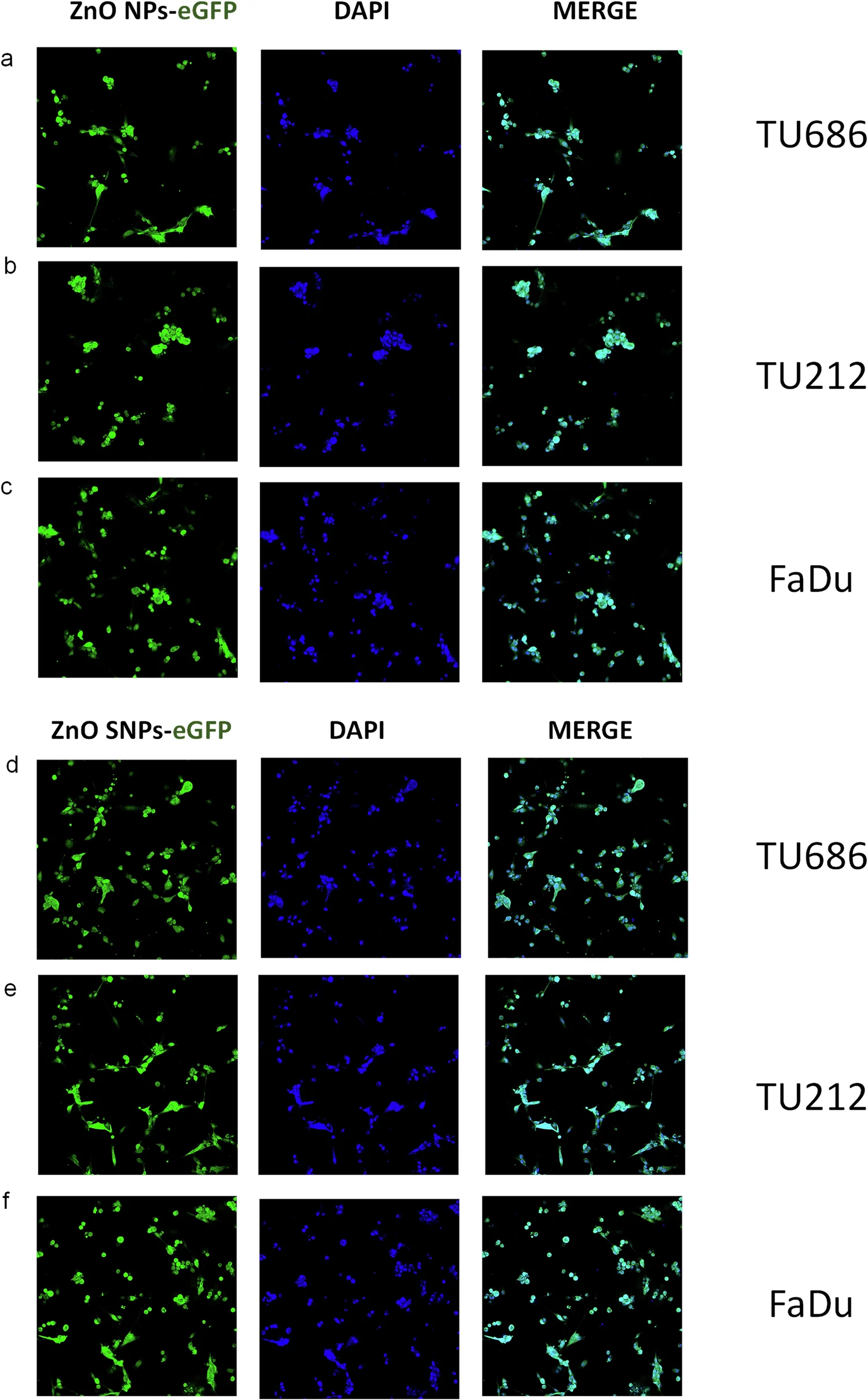
Presence of eGFP-ZnO NPs and SNPs with various particle sizes in HNSCC cell lines. eGFP-ZnO NPs and SNPs (green) treated TU686, TU212 and Fadu, and nuclei were stained by Hoechst 33342 (blue). D) Cells were imaged using confocal microscopy.

After ZnO NPs treatment, Fadu, TU686 and TU212 showed higher proportion of cell cycle arrest in G0/G1 phase (55.73% vs. 50.39%; 66.67% vs. 47.74% and 65.46% vs. 44.76%, respectively; in treatment vs. in control; all *p* < 0.0001) (Fig. 3d–f). In addition, the proportion of cells in G2/M phase was significantly lower in treatment group than in the control group respectively (all *p* < 0.0001). This finding suggested that the nanoparticles interfered with G0/G1 phase cell cycle arrest, as evidenced by a drop in the percentage of cells in the G2/M stages of the cell cycle. Additionally, a higher proportion of G0/G1 cell cycle arrest was observed in ZnO SNPs treatment group (73.66% for Fadu; 70.73% for TU686 and 71.35% for TU212) comparing to ZnO NPs group (*p* < 0.0001). Thus, ZnO SNPs had a larger impact than ZnO NPs on inducing G0/G1 cell cycle arrest in aggressive HNSCC cell line (Fadu) (Fig. 3d). After 48 h of ZnO NPs and ZnO SNPs treatment, 3D spheroids significantly turned into cell aggregates and lose their spherical properties after ZnO SNPs treatment but not ZnO NPs (scale bar = 200 um) (Fig. 3d–f). The effect of ZnO SNPs inhibiting 3D spheroid formation was higher in aggressive cell line (Fadu) compared to ZnO NPs (Fig. 3d). Figure 4 revealed the cellular internalization of eGFP-tagged ZnO NPs and ZnO SNPs in FaDu, TU686 and TU212 with different particle sizes in nucleus with FaDu having the highest cellular uptake of both nanoparticles. Our results imply that the both ZnO NPs and ZnO SNPs were capable of entering the cell nucleus in all sizes.

**Fig. 5:**
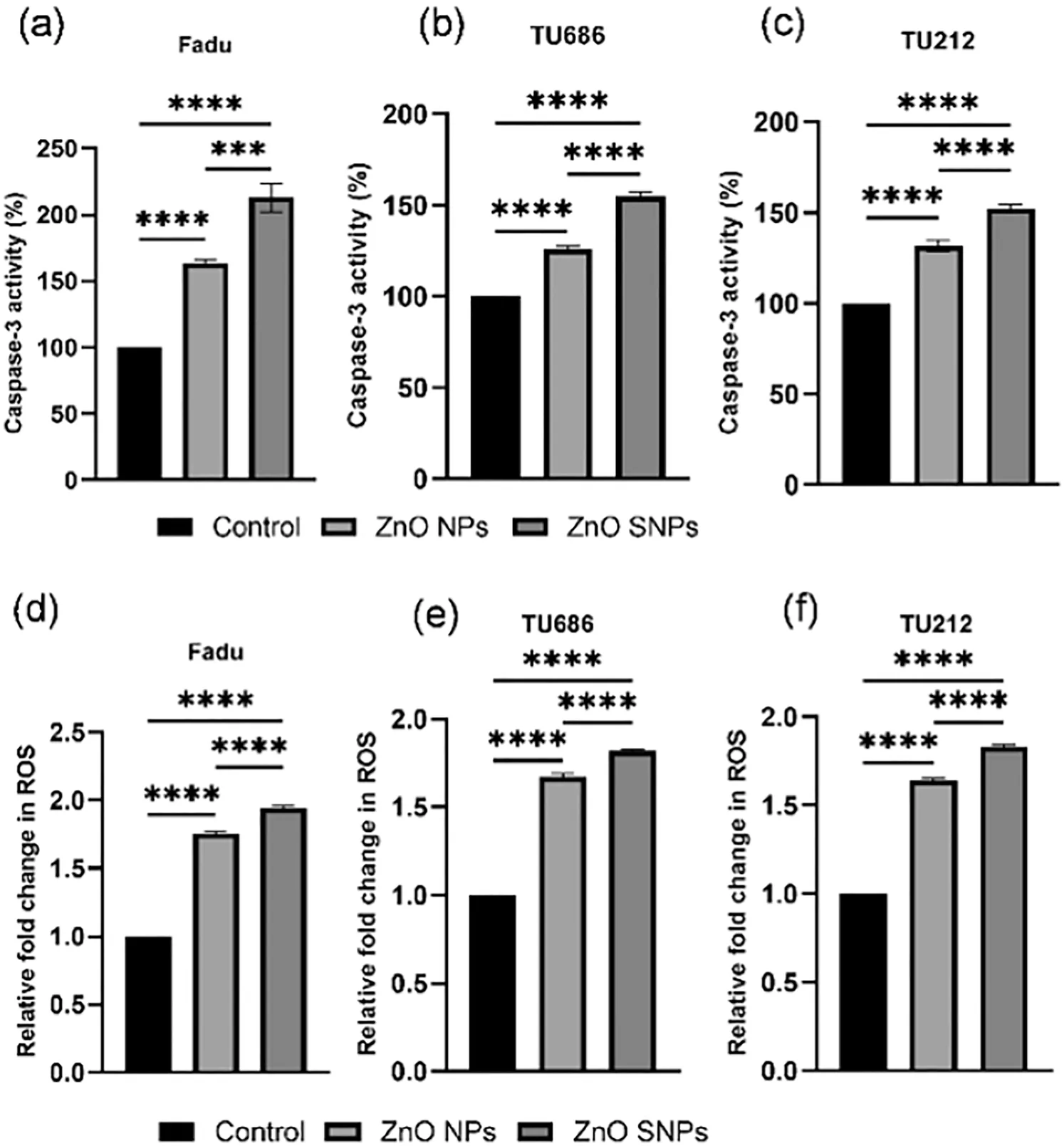
Apoptosis and Reactive Oxygen species evaluation after ZnO NPs and ZnO SNPs treatment. Caspase-3 activity was measured in three HNSCC Fadu **a**, TU686 (**b**) and TU212 (**c**) post ZnO NPs and ZnO SNPs treatment at 405 nm. Intracellular ROS fluorescence intensity in Fadu **d**, TU686 (**e**) and TU212 (**f**) at 525 nm and 580 nm after treated with ZnO NPs and ZnO SNPs for 48 h. All data are expressed as the mean ± standard deviation (SD) from three independent experiments. Ordinary one-way ANOVA was used to determine the significance of the comparisons of data (**** *p* < 0.0001; *** *p* < 0.001; ** *p* < 0.01; *p < 0.05; ns, no significant difference).

Caspase-3 activity was significantly induced by both ZnO NPs and ZnO SNPs leading to apoptosis in HNSCC cell lines compared to control (all *p* < 0.0001). (Fig. 5). However, the effect is more significant in aggressive HNSCC cell line which is Fadu (163.52% in ZnO NPs vs. 212.78% in ZnO SNPs; *p* < 0.0001 (Fig. 5a). This also indicates that ZnO SNPs yielded higher effect in inducing apoptosis through caspase-3 activity in aggressive HNSCC cell line.

Our previously synthesized and characterized Zn SNPs using total polyphenol as reducing reagents exhibited more shift in key peaks in FTIR compared to ZnO NPs. According to XDR results, ZnO SNPs illustrated well-defined peaks indicating purer and organized ZnO phase. Under TEM, clear difference in nano-structure between ZnO NPs and ZnO SNPs was observed where ZnO NPs are spherical and ZnO SNPs showed spike structure. Not only that, our study unveiled the extraction, purification and incorporation of eGFP with ZnO NPs and ZnO SNPs from aggressive glioblastoma cell line (U87) for the first time. Overall, our results revealed inhibitory effect of ZnO NPs and ZnO SNPs HNSCC cell lines especially hypopharyngeal cancer cell line (Fadu). As expected, HNSCC cell lines responded better to ZnO SNPs with lower IC_50_.

In addition, Supplementary Figure 2. Cell viability of ZnO NPs and ZnO SNPs. revealed that ZnO NPs and ZnO SNPs have higher IC50 in HaCaT which is the immortalized cell lines (41.13 ug/ml for ZnO NPs and 36.68 ug/ml for ZnO SNPs) indicating safety profile for normal cell line.

These results agreed with the findings and who reported that the treatment with ZnO NPs and ZnO SNPs suppressed the cancer proliferation cells and they showed minimal effect on normal human cells [14, 20–22]. Moreover, after treatment of ZnO NPs and ZnO SNPs, cell cycle arrest in G0/G1 phase unveiling that this arrest can be caused by a variety of methods, including the generation of oxidative stress, DNA damage, and modification of cell cycle regulatory proteins and it is supported by previous reports that showed that nanoparticles like iron induce G0/G1 arrest [23–26]. Our novel results from the 3D spheroid model also demonstrated that ZnO SNPs decrease the capacity of cancer cells to form 3D structures compared to ZnO NPs and even change them into cell aggregates, which was not disclosed in prior studies. The earlier work by Dang and team demonstrated the effect of ZnO SNPs in several cancer cells, but not in thyroid and prostate cancer, and they failed to employ our 3D spheroid model to standardize their cancer model [14].

### ZnO SNPs indirectly induce ferroptosis via inhibition of GPX4 activity, ROS production and MRP1 gene in HNSCC cell lines

Cells treated with ZnO NPs and SNPs for 48 h showed increased ROS generation relative to the control group (all *p* < 0.0001) (Fig. 3d). In aggressive cell line, ZnO SNPs were shown to be more efficient in boosting ROS generation than ZnO NPs (1.75 vs. 1.94 in Fadu, 1.67 vs. 1.82 in TU686 and 1.64 vs. 1.83 in TU212; ZnO vs. ZnO SNPs; *p* < 0.0001). Therefore, the results demonstrate that ZnO NPs and ZnO SNPs treatment may effectively regulate ROS generation in HNSCC cell lines. ROS production in ferroptosis is inhibited by GPX4 activity; thus, we investigated the ability of ZnO SNPs to indirectly induce ferroptosis through inhibition of GPX4 activity. Figure 4 revealed the cellular internalization of eGFP-tagged ZnO NPs and ZnO SNPs in FaDu, TU686 and TU212 with different particle sizes in nucleus with FaDu having the highest cellular uptake of both nanoparticles. Our results imply that the both ZnO NPs and ZnO SNPs were capable of entering the cell nucleus in all sizes.

**Fig. 6:**
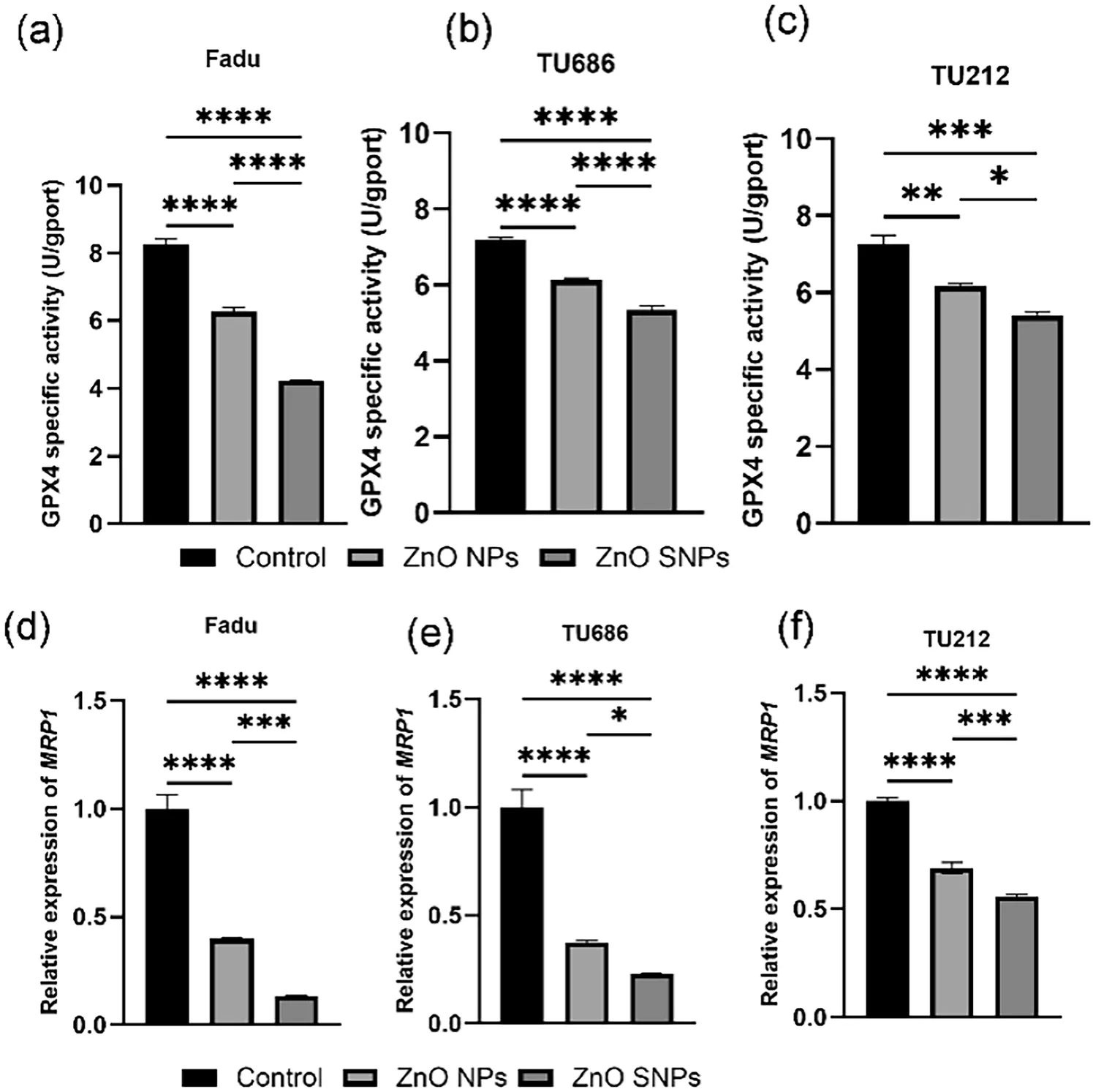
GPX4 specific activity and Relative expression of *MRP1*. Quantification of GPX4 specific activity in Fadu **a**, TU686 (**b**) and TU212 (**c**) following treatment with ZnO NPs and ZnO SNPs measured OD values at 340 nm. Quantitative analysis of relative expression of *MRP1* in Fadu **d**, TU686 (**e**) and TU212 (**f**) by RT-qPCR. GAPDH was used as an internal reference. The relative expression levels of genes were calculated as the method of the 2^−ΔΔCt^. Data are presented as mean ± S.D. Statistical significance was determined by one-way ANOVA. Statistical values are the mean ± standard deviation (SD; vertical bars) of three independent experiments. data (**** *p* < 0.0001; *** *p* < 0.001; ** *p* < 0.01; *p < 0.05; ns, no significant difference)

After ZnO SNPs treatment, GPX4 activity was measured in HNSCC cell lines and results showed that ZnO SNPs can inhibit GPX4 activity thereby promoting ferroptosis in HNSCC cell lines particularly in Fadu cell line (8.24 U/gport vs. 6.26 U/gport vs. 4.24 U/gport; control vs. ZnO NPs vs. ZnO SNPs respectively; *p* < 0.0001) (Fig. 6a). In TU686 and TU212, inhibition of GPX4 activity by ZnO SNPs is less effective compared to ZnO NPs (*p* < 0.05); ZnO NPs vs. ZnO SNPs) (Fig. 6b–c). To investigate the impact of ZnO SNPs treatment on Multidrug resistance-associated protein 1 in HNSCC cell lines, we performed Quantitative real‑time PCR (qRT‑PCR) on *MRP1*, a gene involved in drug-resistant in cancer. It was found that the level of expression of *MRP1* is lower after ZnO SNPs treatment. This unveiled that ZnO SNPs can potentially reverse *MRP1* expression leading to lower drug-resistant signature in HSNCC cell (all *p* < 0.05) (Fig. 6d–e). We have investigated that ZnO SNPs can modulate ROS activity. In supplementary Figure 4, GPX4 KO reduces the expression of MRP1 showing direct relationship between these two onco-proteins.

**Fig. 7:**
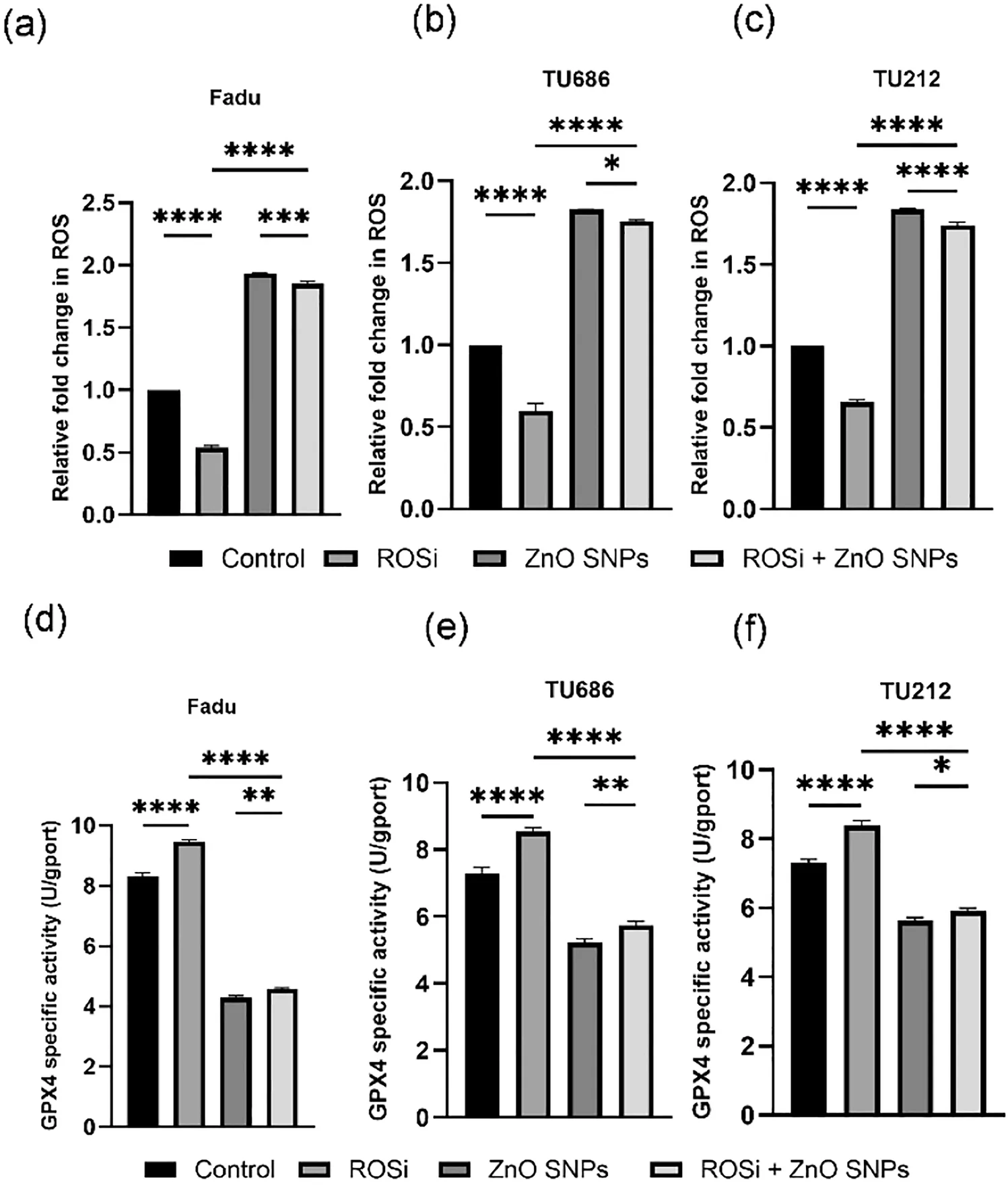
The relative fold change of intracellular ROS and GPX4 in three HNSCC cell lines after incubated with overdose concentration of Histamine dihydrochloride. Quantification of ROS in Fadu **a**, TU686 (**b**) and TU212 (**c**) was determined by fluorescence intensity at 525 nm and 580 nm after treated with ZnO SNPs for 48 h showing reverse effect of ZnO SNPs on ROS inhibitor (1.0 mg/ml). Quantification of GPX4 specific activity in Fadu **d**, TU686 (**e**) and TU212 (**f**) after incubated with ROS inhibitor following treatment with ZnO NPs and ZnO SNPs measured OD values at 340 nm. All data are expressed as the mean ± standard deviation (SD) from three independent experiments. Ordinary one-way ANOVA was used to determine the significance of the comparisons of data (**** *p* < 0.0001; *** *p* < 0.001; ** *p* < 0.01; *p < 0.05; ns, no significant difference)

As shown in Fig. 7a–c, ZnO SNPs can actively reverse the effect of Histamine Dihydrochloride. in HNSCC cell lines compared to the ROSi control group and the effect more prominent in Fadu which is aggressive hypopharyngeal cancer cell line (0.54 vs. 1.85; ROSi vs. ROSi + ZnO SNPs; *p* < 0.0001). TU686 and TU212 showed similar trend respectively (0.6 vs. 1.75 for TU686 and 0.66 vs. 1.74; ROSi vs. ROSi + ZnO SNPs; *p* < 0.0001). To determine the association of ZnO SNPs and inhibition of GPX4 after adding ROS inhibitor. The results revealed that ROS inhibition increased GPX4 activity HNSCC cell lines as expected (all *p* < 0.0001) (Fig. 7d–f). Interestingly, enhanced activity of GPX4 was reverse by ZnO SNPs treatment across three HNSCC cell lines and the effect was more pronounced in hypopharyngeal cancer cell line, Fadu (8.30 vs. 9.46; control vs. ROSi; *p* < 0.0001) (9.46 vs. 4.57; ROSi vs. ROSi + ZnO SNPs; *p* < 0.01) (Fig. 7d).

Ferroptosis in HNSCC is frequently produced by the buildup of lipid peroxidation and reactive oxygen species, and it has been associated to tumor cell growth, metastasis, and therapy resistance [27]. Moreover, our study showed that these two compounds induced cell cycle arrest in G0/G1 phase, promote apoptosis and ROS production as well as decreased GPX4 activity and 3D cell aggregation in HNSCC cell lines. Not only that, in our study, under the effect of ROS inhibitor, ZnO SNPs can reverse its effects by elevating ROS production and decreasing GPX4 activity as well *MRP1* expression.

Our study revealed that treatment with ZnO NPs and ZnO SNPs can induce ROS production and inhibit GPX4 activity indicating that our synthesized particles have potential to regulate cancer cell ferroptosis through ROS-GPX4 axis. This is in accordance with the previous studies since GPX4 play role in inhibiting ferroptosis and its inhibition enhances drug-sensitivity in cancer [28, 29]. *MRP1* encodes the multidrug resistance protein 1, contributes to treatment resistance in HNSCC by facilitating drug efflux from cancer cells [30]. Elevated oxidative stress in cancer cells can lead to ferroptosis, which can enhance the efficiency of chemotherapy or radiation [31, 32]. While the significance of MRP1 in HNSCC is less well explored than in other cancers, its existence and function are important for grasping drug resistance pathways in this kind of cancer [27]. Our study illustrated that ZnO NPs and ZnO SNPs can decrease expression of *MRP1* decreasing resistant-signature in aggressive HNSCC cell line. These tactics provide intriguing new approaches to treating multidrug-resistant feature using ZnO SNPs that go beyond basic MRP1 reversal and provide fascinating avenues for future in vivo and clinical study in HNSCC.

The interplay between GPX4, ROS, ferroptosis, MRP1, and ZnO nanoparticles presents a complex but intriguing landscape in cellular oxidative stress and death mechanisms. GPX4 (glutathione peroxidase 4) plays a crucial role as a key regulator of ferroptosis, an iron-dependent form of regulated cell death characterized by the accumulation of lipid peroxides. By reducing lipid hydroperoxides, GPX4 helps maintain cellular membrane integrity and prevents oxidative damage [33]. When GPX4 activity is compromised, either through genetic knockdown or oxidative stress, ROS (reactive oxygen species) levels tend to increase, leading to enhanced lipid peroxidation and triggering ferroptosis [34, 37]. Interestingly, MRP1 (multidrug resistance-associated protein 1), an ATP-binding cassette transporter, can influence cellular responses to oxidative stress by exporting glutathione conjugates and other antioxidants, thereby modulating ROS levels and potentially affecting ferroptosis susceptibility [7, 35]. Zinc oxide (ZnO) nanoparticles are known to induce ROS making promising agents in cancer therapy, where the induction of ferroptosis could be exploited to selectively kill tumor cells [6, 36]. Our study showed the impact of ZnO nanoparticles on GPX4 activity and MRP1 expression in ferroptosis. While they can induce oxidative damage leading to ferroptosis, cells may respond by upregulating MRP1 to export oxidized glutathione and other toxic compounds, potentially conferring resistance. Therefore, understanding the balance between ROS generation by ZnO nanoparticles, GPX4-mediated detoxification, and MRP1-mediated efflux is essential for optimizing nanoparticle-based therapies. Overall, targeting these pathways could enhance the efficacy of nanomaterials in cancer treatment, but it also underscores the importance of carefully modulating oxidative stress responses to prevent unwanted toxicity.

For the first time, this study unveiled anti-cancer effects and underline mechanisms of our newly developed ZnO NPs and ZnO SNPs using total polyphenol (TP) as a reducing and stabilizing agent in HNSCC. We unveiled the ferroptosis-related axis in our discovery indicating potential avenue in HNSCC therapy model aiming to enhance treatment strategy and lower side effects from cancer treatment. In addition, these nano composites can be further applied in the field of drug delivery in the future with mechanistic and in vivo validation.

In Supplementary Figure 3, lipid peroxidation study showed that both ZnO NPs and ZnO SNPs induced lipid peroxidation compared to the control in HNSCC (all *p* < 0.05). Moreover, after 5 uM of Ferrostatin-1 (Fer-1) induction, ZnO SNPs revealed higher effect of lipid peroxidation showing ZnO SNPs targeting ferroptosis. Moreover, the results showed that after GPX4 knockdown, the expression of MRP1 is relatively lower indicating that HNSCC cell lines with low GPX4 will have lower profile of multidrug resistance (Supplementary Figure 4). In addition, supplementary Figure 6 illustrated that there is no histopathological change in major organs (Spleen, Kidneys and Liver) after ZnO SNPs treatment showing safety profile in vivo. Finally, supplementary figure 5 illustrated that ZnO NPs and SNPs can reduce tumor size in vivo in most aggressive cell line (FaDu) showing anti-tumor effect in mice after 14 days of administration with better effect of SNPs.

## Conclusions

In summary, this study delineates the novel and critical impact of green-synthesized ZnO NPs and ZnO SNPs on ferroptosis and tumor metabolic activity within HNSCC. Our findings demonstrate that these nanoparticles effectively modulate the MRP1/ROS/GPX4 signaling axis, leading to enhanced cytotoxic efficacy. Rigorous in vitro and in vivo evaluations confirmed a favorable safety profile, underscoring the potential of these nanoplatforms for clinical application. However, direct validation of ferroptosis pathway should be further investigated in future studies. Notably, the unique morphological advantages of ZnO SNPs suggest they may serve not only as intrinsic therapeutic agents but also as versatile carriers to improve the delivery and efficacy of conventional treatments. Furthermore, the development of these cell line-derived, fluorescence-guided nanoparticles presents a dual-purpose strategy for both targeted therapy and advanced diagnostics in oncology. While these results are promising, future research utilizing high-resolution, real-time in vivo imaging will be essential to fully validate the mechanistic pathways and establish the translational significance of ZnO SNPs in the management of Head and Neck Squamous Cell Carcinoma.

## Supplementary information

Supplementary Material 1

Supplementary material 2

## Data Availability

All data needed to support the conclusions in the paper are presented in the manuscript and/or the Electronic Supplementary Material. Additional data related to this paper may be requested from the corresponding author upon request.
