# Supplementary material for: eGFP tagged ZnO nanoparticles from glioblastoma targeting ferroptosis and MRP1 in head and neck squamous cell carcinoma
Source: Discov Nano. 2026 Sep 14;21(1):438. doi: 10.1186/s11671-026-04895-x (PMC13575049; doi:10.1186/s11671-026-04895-x)

Supplementary Information (Raw western Blot images)

1. GPX4

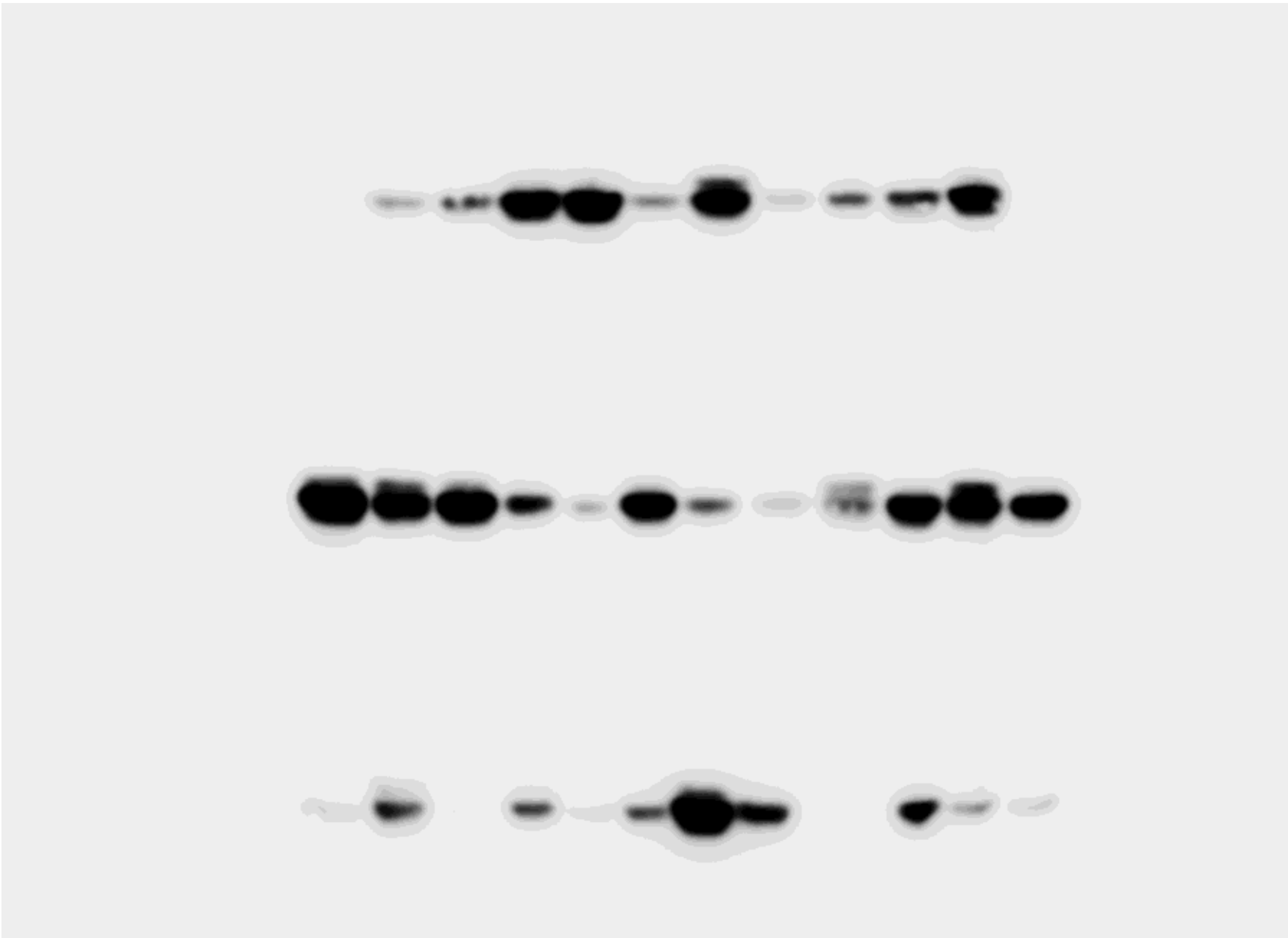

2. MRP1

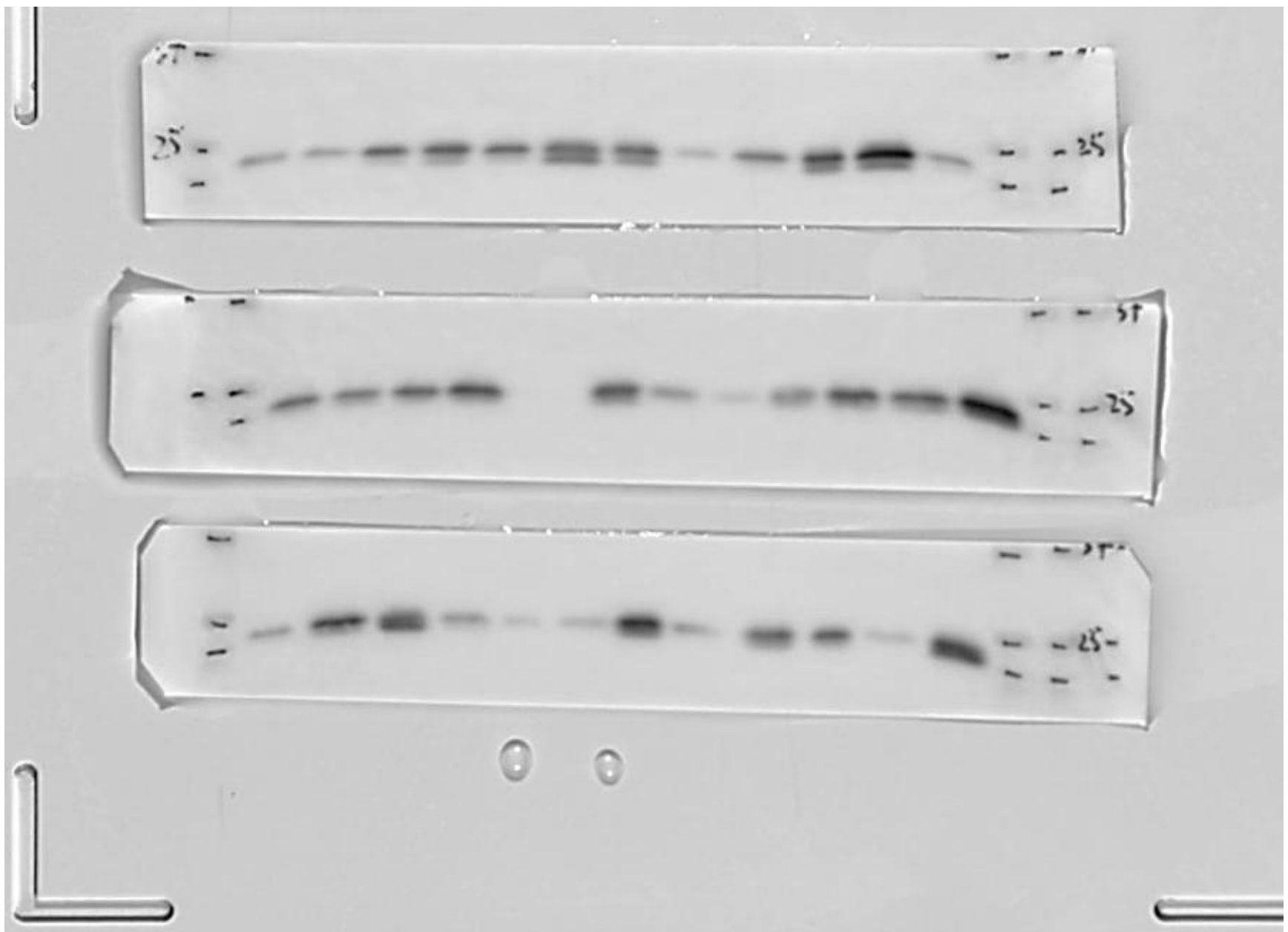

3. Beta actin

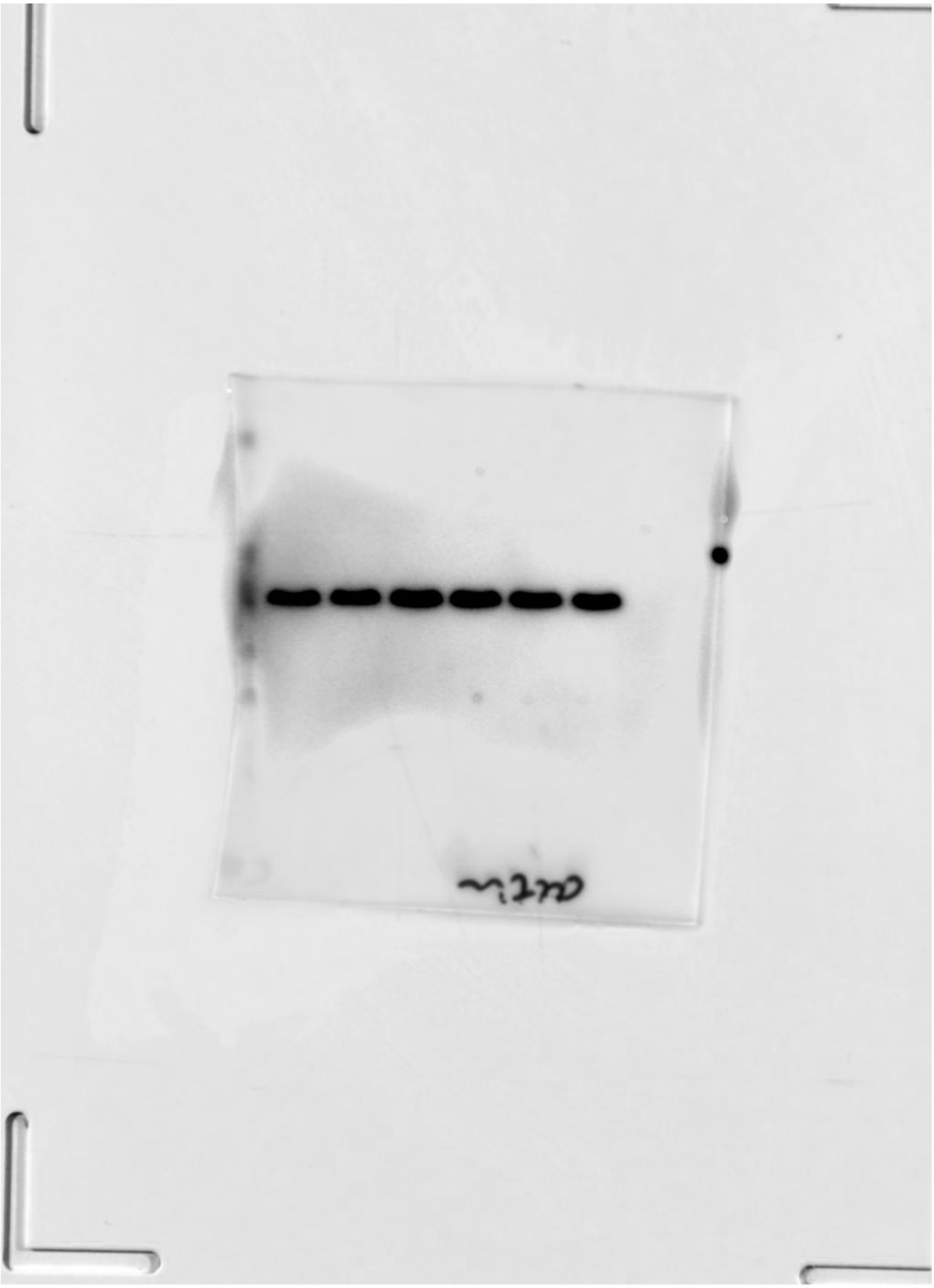

Supplement: Supplementary file 1 [file 11671_2026_4895_MOESM2_ESM.pdf]
