# Supplementary material for: eGFP tagged ZnO nanoparticles from glioblastoma targeting ferroptosis and MRP1 in head and neck squamous cell carcinoma
Source: Discov Nano. 2026 Sep 14;21(1):438. doi: 10.1186/s11671-026-04895-x (PMC13575049; doi:10.1186/s11671-026-04895-x)
Supplement: Supplementary file 2 [file 11671_2026_4895_MOESM1_ESM.docx]

Electronic Supplementary Material

**eGFP tagged ZnO Nanoparticles from glioblastoma targeting Ferroptosis and MRP1 in Head and Neck Squamous Cell Carcinoma**

*Salida Ali^1#^, Yu Li^2#^, Ontana Yotnarong^3#,^ Chi Yao^1^, Yongle Zhan^1^, Ruochen Ma^1^, Ruofan Shi^1,4^, Zhiqiang Wang^2*^, Rong Na^1*^, Theeranan Tangthong^3^**

*^1^Division of Urology, Department of Surgery, School of Clinical Medicine, LKS Faculty of Medicine, The University of Hong Kong, Hong Kong, China.*

*^2^ Department of Otolaryngology-Head and Neck Surgery, The Eighth Affiliated Hospital, Sun Yat-sen University, Shenzhen 518033, China*

*^3^ Nuclear Technology Research and Development Center, Thailand Institute of Nuclear Technology (Public Organization), Nakhon Nayok, Thailand*

*^4^Department of Urology, Ruijin Hospital, Shanghai Jiao Tong University School of Medicine, Shanghai, China.*

\# Salida Ali, Yu Li and Ontana Yotnarong contributed equally to this work

Address correspondence to theeranan@tint.or.th, yungna@hku.hk and [wangzqx@163.com](mailto:wangzqx@163.com)

**ABSTRACT:**

**Background:** Head and neck squamous cell carcinoma (HNSCC) remain one of the most aggressive malignancies worldwide, necessitating the development of targeted therapeutic strategies. While zinc oxide (ZnO) nanostructures have shown promise in nanomedicine, the mechanistic influence of nanoparticle morphology on HNSCC remains poorly understood.

**Methods:** In this study, we synthesized and characterized green-synthesized spherical ZnO nanoparticles (NPs) and spiky ZnO nanoparticles (SNPs) using total polyphenols (TP) as a reducing and stabilizing agent. To track cellular interactions, the nanostructures were further modified with glioblastoma-derived eGFP tags. The antitumor efficacy and underlying mechanisms were evaluated across multiple HNSCC cell lines (Fadu, TU212, and TU686) using 2D cultures, 3D multicellular spheroids, and cellular internalization assays.

**Results:** Chemical characterization revealed distinct architectural shifts between the spherical and spiky morphologies. Both nanoparticle types demonstrated significant dose-dependent anticancer activity; however, ZnO SNPs exhibited superior internalization and more pronounced tumor suppression in 3D spheroid models. Notably, ZnO SNPs showed higher efficacy in the highly aggressive hypopharyngeal cancer line (Fadu) compared to laryngeal carcinoma lines (TU212 and TU686).

Conclusion: Mechanistic investigations revealed that ZnO SNPs exert their antitumor effects by modulating reactive oxygen species (ROS) production, downregulating multidrug resistance-associated protein 1 (MRP1), and inhibiting glutathione peroxidase 4 (GPX4). This synergy triggers potent ROS-induced ferroptosis. Our findings suggest that the hierarchical morphology of ZnO SNPs enhances their therapeutic potential, offering a novel, targeted approach for the treatment of aggressive HNSCC.

**Supplementary methods**

**Materials**

A commercial green tea powder (GT) was purchased from AP Operations Co., Ltd. (Chonburi, Thailand). Sodium hydroxide (NaOH) was purchased from RCI Labscan (Thailand), 1-Ethyl-3-(3ʹ-dimethylamino propyl) carbodiimide (EDC) was purchased from TCI Co., Ltd. (Japan). N-Hydroxysuccinimide (NHS) was obtained from Tokyo Kasei Kogyo Co., Ltd. (Japan).

**Methods**

**ZnO NPs and ZnO SNPs synthesis.** Both ZnO NPs and ZnO SNPs were kindly given by our collaborator Dr. Theeranan Tangthong and her team from Nuclear Technology Research and Development Center, Thailand Institute of Nuclear Technology (Public Organization), Nakhon Nayok, Thailand. The detailed methods of synthesis and characterization were published in this study [11] [18].

Materials:

Green tea (Camellia sinensis) extract powder was obtained from AP Operations Co., Ltd. (Chonburi, Thailand). Zinc acetate dihydrate was purchased from Merck (Darmstadt, Germany). Throughout all experiments, distilled water was used. Gallic acid was sourced from Sisco Research Laboratories Pvt. Ltd. (Maharashtra, India), and Folin-Ciocalteu reagent was also supplied by the same company.

Preparation of Total Phenols (TP) as Reducing and Stabilizing Agents:

Green tea powder was irradiated with gamma rays using a 60Co source (Gamma Cell 220 irradiator) at a dose rate of 8.11 kGy/h, delivering a total dose of 10 kGy at ambient temperature in air. Post-irradiation, the irradiated green tea (GT-10) powder was used to produce polyphenols. To extract TP, 60 mg of GT-10 powder was dissolved in 10 mL of deionized water and mixed with an equal volume of 50% ethanol (w/v). The mixture was incubated at room temperature for 25 minutes, then centrifuged at 13,000 rpm and 4°C for 10 minutes. The supernatant was discarded, and the pellet was dried at 60°C for 48 hours. The extracted TP was characterized by UV-Vis spectroscopy to confirm its composition.

Determination of Phenol Content:

The total polyphenol content (TPC) was quantified using the Folin-Ciocalteu method. Briefly, 2.3 mg of Folin-Ciocalteu reagent was dissolved in 10 mL deionized water. Extracted polyphenols from green tea leaves were mixed with the reagent in a 1:1 (v/v) ratio, vortexed, and incubated in the dark at room temperature for 2 hours. Gallic acid (GA) standards at various concentrations were prepared similarly for calibration. Absorbance was measured at 765 nm using UV-Vis spectroscopy. The highest TPC observed was 0.42 mg/mL.

Green Synthesis of ZnO NPs and ZnO SNPs:

Briefly, we have conducted green synthesis of ZnO nanoparticles (ZnO NPs) using total phenols (TP) as reducing agent and stabilizing agent from green tea (Camellia sinensis). Green synthesis of ZnO NPs were prepared by using the precursor of 0.2 M zinc acetate dihydrate ([Zn(CH₃COO)₂·2H₂O] solution mixed with the reducing agent of 1 mg/ml of TP solution at a ratio 1:1, and kept mixed-solution at room temperature for 9 hrs under dark conditions. Then, they chemically synthesized ZnO SNPs using zinc acetate dihydrate [Zn(CH₃COO)₂·2H₂O] and sodium hydroxide (NaOH) as precursor materials. 0.06 M of zinc acetate dihydrate [Zn(CH₃COO)₂·2H₂O] was dissolved in 50 mL distilled (DI) water, and 0.12 M NaOH was added slowly drop by drop with vigorous stirring. Then, the mixture solution was stirred for 5 hrs. Afterward, the aqueous precipitate was washed three times with deionized water, transferred to a crucible, and calcined at 450 °C for 5 hrs in an annealing furnace. The resulting ZnO SNPs powder was then collected and stored in a drying oven for subsequent use.

**Extraction and Purification of eGFP from Glioblastoma cell line:**

The eGFP-tagged U87 was kindly given by Prof. Gilberto Leung from Department of Surgery, The University of Hong Kong. After the trypsinization of the cell, the cells were washed with PBS and the 10 ul of the proteinase and phosphatase inhibitor cocktail was added to the 990 ul of RIPA buffer to achieve 1X solution (on ice). Then, the cells were centrifuged at 15 000 × g for 15 min and the supernatant was collected and measured the protein concentration. The purification was done using Immobilized Metal Affinity Chromatography (IMAC) method which will selectively capture histidine-tagged proteins like EGFP. After that, the absorbance at 280 nm for protein and 488 nm for EGFP were used to quantify protein concentration and purity.

Preparation of eGFP- ZnO NPs and ZnO SNPs:

The 10 ul of aminopropyltriethoxysilane (APTES) in toluene was used to introduce amine group to ZnO NPs and ZnO SNPs. Then, the 10 ul of crosslinker EDC (1-ethyl-3-(3-dimethy laminopropyl) carbodiimide) and NHS (N-hydroxysuccinimide) were used to activate carboxyl groups of eGFP. This step was carried out in MES buffer at pH 5.5. Then, both eGFP and ZnO NPs and ZnO SNPs were mixed in room temperature overnight with constant stirring. Then, the confocal microscope was used to detect eGFP- ZnO NPs and ZnO SNPs.

**Preparation of eGFP- ZnO NPs and ZnO SNPs:**

The 10 ul of aminopropyltriethoxysilane (APTES) in toluene was used to introduce amine group to ZnO NPs and ZnO SNPs. Then, the 10 ul of crosslinker EDC (1-ethyl-3-(3-dimethylaminopropyl) carbodiimide) and NHS (N-hydroxysuccinimide) were used to activate carboxyl groups of eGFP. This step was carried out in MES buffer at pH 5.5. Then, both eGFP and ZnO NPs and ZnO SNPs were mixed in room temperature overnight with constant stirring. Then, the confocal microscope was used to detect eGFP- ZnO NPs and ZnO SNPs.

**Cellular internalization of eGFP - ZnO NPs and ZnO SNPs in HNSCC cell lines:**

eGFP- ZnO NPs and ZnO SNPs were used to treat three HNSCC cell lines including laryngeal carcinoma cell lines (TU686 and TU212) and hypopharyngeal cancer cell line (Fadu) for 48 hrs and cellular internalization were observed using confocal Microscope LSM 880 with Airyscan 1.

**Preparation of ZnO NPs and ZnO SNPs for cellular phenotypes experiment:**

The whole phenol extracts and Zn acetate produced using prior procedures were dissolved individually in 3ml of deionized water (vortex violently). The TP extract solution and Zn acetate solution were then gently combined in a light-protected container (vortex violently for 10 seconds). The combination was then incubated at room temperature (in a dark setting) for 12-15 hours to produce ZnO NPs solution. Following incubation, the dark brown precipitate confirmed the presence of ZnO NPs. The mixture was then swirled for 30 minutes or sonicated at 25°C for 15 minutes to ensure that the particles were well mixed. The solution of ZnO NPs was diluted at ranges of concentrations (0, 6.25, 12.5, 25, 50, and 100 μg/mL) before the cellular experiment. To conduct cellular experiments, dried ZnO SNPs powder was diluted in deionized water at doses of 0, 6.25, 12.5, 25, 50, and 100 μg/mL.

***In vitro* validation**

**Cell culture.** Three HNSCC cell lines including laryngeal carcinoma cell lines (TU686 and TU212) and hypopharyngeal cancer cell line (Fadu) (Chinese Academy of Sciences Cell Bank in Shanghai, China) were cultured in Roswell Park Memorial Institute medium (RPMI 1640) (Gibco, USA) with 10% heat-inactivated fetal bovine serum (FBS) and 1% antibiotic-antimycotic (ThermoFisher Scientific). All cell lines were incubated in a humidified incubator at 37 °C with 5% CO_2_ supply. They were passaged once every 2–3 days using trypsin and PBS. Mycoplasma contamination was performed throughout the cell culture process.

**Methyl Thiazolyl Tetrazolium (MTT) Assay.** Cytotoxicity evaluation of ZnO NPs and ZnO SNPs was performed using Cell Proliferation Kit I (MTT) (Roche, Switzerland). Cells were seeded at a population density of 2000 cells/well for FaDu and 4000 cells/well for TU686 and TU212 respectively in 96-well plates. Different concentrations of ZnO NPs and ZnO SNPs (0, 6.25, 12.5, 25, 50 and 100 μg/mL) were used as treatment for 48 hrs. After 48 hours of incubation, 10 μl of MTT reagent was added to each well and was further incubated for 4 hours. Then, 100 μl solubilizing reagent was added and incubated in a humidified atmosphere overnight. Absorbance of the formazan product at 570 nm was measured by Thermo Varioskan flash reader with a reference wavelength at 650 nm. GraphPad Prism 10 was used as a tool for statistical analysis to determine percent cell viability and half-maximal inhibitory concentration (IC_50_) of ZnO NPs and ZnO SNPs. The experiment was performed in both internal and external triplicates.

**Cell cycle analysis.** Flow cytometry was utilized to analyze the percentage of each cell cycle phase (G1, S and G2/M). After the treatment, cells were collected and fixed with 70% cold ethanol at 4 °C for 1h and were centrifuged at 1500 g for 5 min and incubated in 0.5ml PBS containing 0.1mg/ml RNase for 30 min at 37 °C. stained with Propidium Iodide (Invitrogen, USA) were added for DNA staining. Data acquisition and analysis were performed in Cell Analyzer Agilent NovoCyte Quanteon. Total event for each sample was set as 1×10^4^ events per 100 ul. NovoExpress software and GraphPad Prism were used as tools the gating and data analysis.

**Caspase-3 activity Assay.** Caspase-3 activity was measured using a Caspase-3 Activity Colorimetric Assay Kit (Elabscience, China), following the manufacturer's instructions. After the treatment, cells were digested with trypsin, and centrifuged at 500 g for 5 minutes before discarding the supernatant. The pellet was reconstituted in pre-cooled Cell Lysis Buffer at a ratio of 100 μL per 1 million cells and incubated on an ice bath for 30 minutes. The lysed samples were centrifuged at 11000×g for 10-15 minutes at 4°C. The supernatant was transferred to a fresh tube and stored on ice until use. To set up the reaction system, 100 μg of protein from the lysate supernatant was utilized, following instructions provided by the manufacturer.

After adding Ac-DEVD.pNA and mixing, the reaction was incubated at 37°C for 2–4 hours. The samples' absorbance levels were then measured using a microplate reader reads at 405 nm. Caspase-3 activity in each group was calculated using the following formula: (OD sample - OD blank)/(OD control - OD blank)*100.

**3D spheroid generation model.** RPMI 1640 (Gibco, USA), N2 supplement (Gibco, USA), B-27™ Supplement (50X) minus vitamin A (Gibco, USA), L-Glutamine (Gibco, USA), 100 ng/ul Recombinant Human Epidermal Growth Factor (EGF) (Gibco, USA), 100 ng/ul Recombinant Human Fibroblast Growth Factor-basic (FGF) (Gibco, USA) are components of complete media. After trypsinization, three cancer cell lines spheroid sizes were standardized using Aggrewell^400^ 24 well plates (Stemcell Technologies). Fadu was seeded with concentration of 1.2 x 10^6^ per well and TU686 and TU212 were seeded at 2.4 x 10^6^ cells per well. Each well was pretreated with 500 µL of Anti-Adherence Rinsing Solution and centrifuged at 1300 x g for 5 minutes. Then, Anti-Adherence Rinsing Solution was aspirated, and wells were washed with 2 mL of warm RPMI 1640 basal medium and added 1 mL of warm complete medium.

**ZnO NPs and ZnO SNPs treatment in cancer spheroids.** After 48 hours of cell seeding, the spheroids were examined under a microscope for uniform size and morphology (scale bar = 200 um), and then treated with half-maximal inhibitory concentrations (IC50) of ZnO NPs and ZnO SNPs for 48 hours. Then the spheroids' size and form were examined. Spheroids are also extracted and used as passageways for other phenotypes.

**Reactive Oxygen Species (ROS) Assay.** The levels of intracellular ROS were examined using Reactive Oxygen Species Assay Kit (Beyotime, China). Cells were incubated with 10 μM H2DCFDA (Beyotime Biotech, China) in the dark for 20-30 min and then washed twice with deionized water. The fluorescence intensity at 525 nm and 580 nm were read using a Microplate Reader (BMG CLARIOstar Plus). The data were calculated as relative fold change of ROS comparing to the control.

**Inhibition of Reactive Oxygen Species (ROS) using overdosed Histamine dihydrochloride****.** Histamine dihydrochloride was shown to have an IC 50 of 0.1 mg/ml against cancer cell types in vitro, showing anti-tumor action. Histamine dihydrochloride, a derivative of histamine, has been shown to suppress the generation of reactive oxygen species (ROS). Thus, in our study, we use overdose concentration (1.0 mg/ml) of Histamine dihydrochloride to inhibit ROS production in each cell line to evaluate if ZnO SNPs can regulate ROS production in hypopharyngeal cancer cell lines (Fadu) and laryngeal carcinoma cell lines (TU212 and TU686)

**Glutathione Peroxidase 4 (GPX4) Activity Assay.** To indirectly quantify ferroptosis via GPX4 activity. GPX4 activity was detected using a Glutathione Peroxidase 4 (GPX4) Activity Assay Kit (Elabscience, China) according to the manufacturer's protocols. Briefly, after treatment and intervention, cells were cultured, digested with trypsin, and centrifuged at 500 g for 5 min before the supernatant was discarded. Cells were washed with PBS and homogenized in 200 µL extract working solution using ultrasonic cell disruptor at 4°C. Then, cells were centrifuged at 10000×g for 10 minutes to remove insoluble material and supernatant was collected and kept on ice for further detection. The supernatant can store at 2-8°C for 1 day. Protein concentration of supernatant was determined. 20 μL of sample and control were added into wells. Then, 40 μL of buffer solution was added into sample well and 40 μL of inhibitor working solution was added into control well. After mixed fully, the plate was incubated at 37℃ for 30 min. Then, 140 μL of reaction working solution and 40 μL of oxidant working solution was added into each well and measured the OD values at 340 nm with microplate reader immediately.

**Quantitative real‑time PCR (qRT‑PCR).** Total RNA was isolated from prostate and thyroid cancer cell lines using a FastPure cell/tissue RNA isolation kit V2 (Vazyme Biotech Co., Ltd., China), according to the manufacturer's instructions. Complementary DNAs (cDNAs) was synthesized with Hifair ® AdvanceFast One-step RT-gDNA Digestion SuperMix for qPCR (Yisheng Biotechnology, China). The qPCR reaction system was prepared using a real-time quantitative PCR instrument according to product specification. GAPDH was used as an internal reference. The relative expression levels of genes were calculated as the method of the 2^−ΔΔCt^. The primers were shown in Table 1.

**Table 1**

| Gene name | Primer Sequences |
| --- | --- |
| *MRP1* | Forward primer:  5′-GGATCATGCTCACTTTCTGG-3’ Reverse primer:  5′-AAGTGATGTCACGAAACAGGTC-3’ |
| *GAPDH* | Forward primer:    5'-TGACTTCAACAGCGACACCCA-3'  Reverse primer:  5'-CACCCTGTTGCTGTAGCCAAA-3' |

**Statistical analyses.** Analyses were performed with GraphPad software 10 (GraphPad Software, CA) from at least three independent experiments, presented as the mean ± standard deviation (SD). The values between two groups were assessed by unpaired two-tailed Student’s t test, and differences among the multiple groups were analyzed by one-way ANOVA and two-way ANOVA, and p value < 0.05 was considered statistically significant. (**** *p* < 0.0001; *** *p* < 0.001; ** *p* < 0.01; **p* < 0.05; ns, no significant difference).

**Supplementary Figure**

**Supplementary figure 1.** (A) The UV-Vis spectra of NPs were compared with Zn^2+^ precursor (blue line) TP reducing agent (orange line) (B) TEM image and (C) size distribution of (a) ZnO NPs and (b) ZnO SNPs.

**Supplementary Figure 2.** Cell viability of ZnO NPs and ZnO SNPs.

Address correspondence to theeranan@tint.or.th, yungna@hku.hk and wangzqx@163.com

**Supplementary Figure 3.** Lipid peroxidation levels measured by malondialdehyde (MDA) assay. The extent of lipid peroxidation was determined by quantifying MDA content in HNSCC cell lines with and without ZnO NPs and ZnO SNPs. The results are expressed as nmol. Data represent the mean ± SD of three independent experiments. Statistical significance was evaluated using ANOVA test, with *p<0.05 and ****p<0.0001 indicating significant differences between groups.

**Supplementary Figure 4.** Western Blot analysis indicating GPX4 knockout with relative expression of MRP1 in HNSCC cell lines

**Supplementary Figure 5.** In vivo validation of ZnO NPs and SNPs treatment effects in FaDu.

**Supplementary Figure 6.** In vivo validation of toxicity of ZnO NPs and SNPs in major organs.
